# LAT2 regulates glutamine-dependent mTOR activation to promote glycolysis and chemoresistance in pancreatic cancer

**DOI:** 10.1186/s13046-018-0947-4

**Published:** 2018-11-12

**Authors:** Mengyu Feng, Guangbing Xiong, Zhe Cao, Gang Yang, Suli Zheng, Jiangdong Qiu, Lei You, Lianfang Zheng, Taiping Zhang, Yupei Zhao

**Affiliations:** 1 Department of General Surgery, Peking Union Medical College Hospital, Chinese Academy of Medical Sciences and Peking Union Medical College, No. 1 Shuaifuyuan, Wangfujing Street, Beijing, 100730 China; 2 Department of Biliary-Pancreatic Surgery, Affiliated Tongji Hospital, Tongji Medical College, Huazhong University of Science and Technology, Wuhan, 430030 China; 3 Department of Nuclear Medicine, Peking Union Medical College Hospital, Chinese Academy of Medical Sciences and Peking Union Medical College, Beijing, 100730 China; 4 Clinical Immunology Center, Chinese Academy of Medical Sciences and Peking Union Medical College, No. 1 Shuaifuyuan, Wangfujing Street, Beijing, 100730 China

**Keywords:** L-type amino acid transporter 2, Mechanistic target of rapamycin, Pancreatic cancer, Chemoresistance, Glutamine metabolism, Glycolysis

## Abstract

**Background:**

Reprogrammed energy metabolism has become an emerging hallmark of cancer in recent years. Transporters have been reported to be amino acid sensors involved in controlling mTOR recruitment and activation, which is crucial for the growth of both normal and tumor cells. L-type amino acid transporter 2 (LAT2), encoded by the SLC7A8 gene, is a Na^+^-independent neutral amino acid transporter and is responsible for transporting neutral amino acids, including glutamine, which can activate mTOR. Previous studies have shown that LAT2 was overexpressed in gemcitabine-resistant pancreatic cancer cells. However, the role of LAT2 in chemoresistance in pancreatic cancer remains uncertain and elusive.

**Methods:**

The effects of LAT2 on biological behaviors were analyzed. LAT2 and LDHB levels in tissues were detected, and the clinical value was evaluated.

**Results:**

We demonstrated that LAT2 emerged as an oncogenic protein and could decrease the gemcitabine sensitivity of pancreatic cancer cells in vitro and in vivo. The results of a survival analysis indicated that high expression levels of both LAT2 and LDHB predicted a poor prognosis in patients with pancreatic cancer. Furthermore, we found that LAT2 could promote proliferation, inhibit apoptosis, activate glycolysis and alter glutamine metabolism to activate mTOR in vitro and in vivo. Next, we found that gemcitabine combined with an mTOR inhibitor (RAD001) could reverse the decrease in chemosensitivity caused by LAT2 overexpression in pancreatic cancer cells. Mechanistically, we demonstrated that LAT2 could regulate two glutamine-dependent positive feedback loops (the LAT2/p-mTOR^Ser2448^ loop and the glutamine/p-mTOR^Ser2448^/glutamine synthetase loop) to promote glycolysis and decrease gemcitabine (GEM) sensitivity in pancreatic cancer.

**Conclusion:**

Taken together, our data reveal that LAT2 functions as an oncogenic protein and could regulate glutamine-dependent mTOR activation to promote glycolysis and decrease GEM sensitivity in pancreatic cancer. The LAT2-mTOR-LDHB pathway might be a promising therapeutic target in pancreatic cancer.

**Electronic supplementary material:**

The online version of this article (10.1186/s13046-018-0947-4) contains supplementary material, which is available to authorized users.

## Background

Pancreatic cancer is one of the most lethal digestive tract malignancies, is difficult to detect early and has a poor prognosis. The 5-year overall survival rate has remained relatively stable, at close to 8%, over the past decade [1, 2]. Chemotherapy has become one of the primary and indispensable therapeutic methods. However, chemoresistance to gemcitabine (GEM), which is the first-line adjuvant therapy for pancreatic cancer, is prevalent and has become one of the key reasons that patients with pancreatic cancer show a poor prognosis. Therefore, elucidating the mechanism of chemoresistance to GEM may contribute to enhancing the effects of therapy and improving the prognosis of pancreatic cancer.

Reprogrammed energy metabolism has become an emerging hallmark of cancer in recent years [3]. Previous studies indicated that most metabolites did not show significant changes in pancreatic cancer cells exposed to GEM; however, chemoresistance to GEM induces metabolic reprogramming [4, 5]. Therefore, metabolic reprogramming must be involved in the regulation of chemoresistance. Glutamine and glucose are the primary nutrient sources for cancer cells and are crucial to the biosynthesis of products such as nucleic acids and nonessential amino acids (NEAAs) [6]. Otherwise, glutamine metabolism is involved in both glycolysis and the tricarboxylic acid (TCA) cycle. In addition, other studies show that glutamine can activate mechanistic target of rapamycin (mTOR) [7] and inhibit apoptosis to induce chemoresistance [8]. Thus, glutamine transport and metabolism might play an important role in chemoresistance in pancreatic cancer.

L-type amino acid transporter 2 (LAT2), encoded by the SLC7A8 gene, belongs to the SLC7 subfamily of the LAT family [9]. LAT2 is a Na^+^-independent neutral amino acid transporter and is responsible for transporting neutral amino acids, including Gln, Gly, Ser, Ala, Thr, Asn, Met, Val, Phe, Tyr, Leu, Ile, Trp and His [9, 10]. LAT2 is upregulated in 9 different cancer types according to Oncomine data [9]; however, few studies have proved its specific role in cancer, and the expression level and role of LAT2 in pancreatic cancer remain uncertain and elusive. In our previous study, we identified a cluster of dysregulated mRNAs associated with GEM chemoresistance via mRNA microarray analysis in the established gemcitabine-resistant pancreatic cancer cell line AsPC-1-GEM(data unpublished). Among these dysregulated mRNAs, LAT2 showed a higher expression level in AsPC-1-GEM cells (≥10-fold change) than in the parental cells. Thus, we presumed that LAT2 might be involved in the development and regulation of chemoresistance in pancreatic cancer. Given the previously described role of LAT2 in glutamine transport and metabolism, we hypothesized that LAT2 could promote GEM chemoresistance in pancreatic cancer cells through glutamine-dependent mTOR activation.

## Methods

### Patients, sample collection and immunohistochemistry (IHC)

Formalin-fixed, paraffin-embedded pancreatic cancer (*n* = 87) and matched paracancerous (*n* = 71) tissue microarrays were obtained from Peking Union Medical College Hospital. None of the patients received neoadjuvant therapy. Tumor staging relies on the 8th edition of TNM system designed by the American Joint Committee on Cancer (AJCC). Follow-up depended on medical records and telephone. The primary end point was overall survival. Mouse anti-human LAT2 monoclonal antibodies (GTX83618, GeneTex, 1:100) and rabbit anti-human LDHB polyclonal antibodies (14824–1-AP, Proteintech, 1:100) were used for the standard immunohistochemical staining procedures. The results of immunohistochemistry were scored by adding the percentage score and the intensity score, which were assessed according to the percentage of positive cells and intensity of staining respectively. The percentage score was classified into 4 grades using the percentage of positive cells (1, < 25%; 2, 25–50%; 3, 50–75%; 4, > 75%). The intensity score was classified into 4 grades using the intensity of stained cells (0, none; 1, weak; 2, moderate; 3, strong). LAT2 and LDHB expression was considered to be low if the total score was equal to or less than the median, and considered high if the score was greater than the median.

### Cell culture

The human pancreatic cancer cell lines MIA PaCa-2 and PANC-1 were donated by Dr. Freiss H (University of Heidelberg, Heidelberg, Germany). MIA PaCa-2 and PANC-1 cells were maintained in Dulbecco’s Modified Eagle’s Medium (DMEM, Logan, UT, USA). All Media were supplemented with 10% fetal bovine serum (FBS, Hyclone, Logan, UT, USA) at 37 °C and 5% CO_2_.

### Plasmids and antibodies

Complementary DNA encoding LAT2 was synthesized and subcloned into the pENTER-FLAG vector (Vigene Bioscience, MD, USA) according to the manufacturer’s instructions. For PANC-1 cell lines that stably overexpressed LAT2, the lentivirus plasmid pLVX-LAT2 was constructed by subcloning the LAT2 coding sequence (Gene ID: 23428) into the modified pLVX-puro backbone (Clontech, CA, USA). The forward primer was 5'-GCGGCTAGCATGGAAGAAGGAGCCA-3', and the reverse primer was 5'-CGCGGATCCGGGCTGGGGCTGCC-3'. The PCR products of LAT2 coding sequence were inserted into the pLVX-puro plasmids with Nhe I and BamH I. The pLVX-LAT2 was packaged into lentiviruses. PANC-1 cells were infected with the pLVX-LAT2 lentivirus, and the cell line was got after 2μg/ml puromycin screening.

Antibodies against LAT2 (GTX83618, GeneTex), LDHB (14824–1-AP, Proteintech), p-mTOR (Ser2448) (5536, Cell Signaling Technology), mTOR (2983, Cell Signaling Technology), PKM2 (4053, Cell Signaling Technology), GAPDH (10494–1-AP, Proteintech), Glutaminase (ab156876, Abcam) and Glutamine synthetase (sc-74430, Santa Cruz Biotechnology) were purchased commercially.

### RNA interference

SiLAT2 and siNC were purchased from RiboBio (Guangzhou, China). The sequences for LAT2 siRNA are listed as below: the forward primer was 5'-GAAGAUGAUGAUGCCAAUUTT-3', and the reverse primer was 5'-AAUUGGCAUCAUCAUCUUCTT-3'. MIA PaCa-2 and PANC-1 cells were transfected with siRNA at 50-100 nM packaged by Lipofectamine 3000(Invitrogen, Carlsbad, CA, USA) in 6-well plates (5 × 10^5^cells/well).

### Lentivirus production

HEK293T cells were cultured in individual 15 cm dishes for each viral construction. When the cells reached approximately 70–80% confluency (~ 1.5 × 10^7^ cells), they were cotransfected with 15 μg of the pLVX-LAT2 plasmid, 15 μg of the psPAX2 plasmid (Addgene #12260, Cambridge, MA, USA) and 10 μg of the pMD2.G plasmid (Addgene #12259, Cambridge, MA, USA) plasmid with Lipofectamine 2000 transfection reagent. At 18 h post-transfection, the medium was replaced with 25 mL of fresh complete medium. The supernatants were harvested at 48 h and 72 h, centrifuged at 1000 rpm for 10 min at 4 °C to remove the cells, and filtered through a 0.45 μm filter to remove the debris. Finally, the supernatant was ultracentrifuged at 120,000×g for 2 h at 4 °C, dissolved in PBS after the removal of the supernatant, and stored at − 80 °C.

### CRISPR KO cell line construct

The target sequence was cloned into the lentiCRISPR v2 backbone (Addgene #52961, Cambridge, MA, USA), and two oligonucleotides were generated after digestion with BsmBI. The design of the target sequences was performed using http://crispr.mit.edu to obtain the gRNA sequence. The LAT2 gRNA forward primer sequence was 5’-CACCGTGACATCGGCCTCGTCGCAC-3′, and the reverse primer sequence was 5’-AAACGTGCGACGAGGCCGATGTCAC-3′. LentiCRISPR-LAT2sgRNA was packaged into the lentiviruses. MIA PaCa-2 and PANC-1 cells were infected with the LentiCRISPR-LAT2sgRNA lentivirus for more than 48 h, and the cell lines were obtained 48 h after screening with 2 μg/ml puromycin. Then, the monoclonal cell lines were screened by identifying those with a knockout of LAT2 protein expression and a disturbance of the target DNA sequence.

### Cell proliferation and growth inhibition assay

At 24 h after transfection, MIA PaCa-2 and PANC-1 cells were plated into 96-well culture plates (3000 cells/well) for cell proliferation assays. All the plates were cultured at 37 °C with 5% CO_2_. For proliferation assays, 10 μL/well cell count kit (CCK-8) reagent was added at 0, 24, 48 and 72 h after plating. After an additional 2.5-h incubation with CCK-8 reagent at 37 °C, the optical density (OD) at the 450-nm wavelength (OD450) was measured using a microplate reader (Wellscan MK3, Thermo/Labsystems, Finland). OD630 served as a reference, and the OD in the blank well was used as the base level. For growth inhibition assays, 4000 cells/well were plated into 96-well culture plates at 24 h after transfection. After incubation for 4–6 h for cell adherence, a gemcitabine (Eli Lilly and Company) concentration gradient from 100 nM to 1 mM or control PBS buffer was added into each well. Cell count kit (CCK-8) reagent (10 μL/well) was added after an additional 48-h incubation at 37 °C. Then, the inhibition rate was calculated as follows: OD = OD450-OD630, inhibition rate = 1-(OD_GEM_-OD_blank_) / (OD_PBS_-OD_blank_).

### Apoptosis assay

MIA PaCa-2 and PANC-1 cells were transfected into 6-well plates and treated with 10 μM gemcitabine twenty-four hours later. After treating for 48 h, the cells were collected and resuspended in binding buffer. Next, the cells were stained with Annexin V-FITC and propidium iodide (PI) (Beyotime, China) according to the manufacturer’s instructions. Analyze was carried out using flow cytometry (FACScan, BD Biosciences, USA).

### Metabolism experiments

The OCR and ECAR of cells were measured with an XF96 Extracellular Flux Analyzer (Seahorse Bioscience, North Billerica, MA, USA). Cells were plated (10,000 cells per well for MIA PaCa-2; 5000 cells per well for PANC-1) in at least triplicate for each condition the day before the experiment. The energy phenotype test and glycolytic rate assay were performed as described in the user guides for the Agilent Seahorse XF Cell Energy Phenotype Test Kit (103325–100, Agilent Technologies) and the Agilent Seahorse XF Glycolytic Rate Assay Kit (103344–100, Agilent Technologies). OCR and ECAR were normalized to the cell number as determined by CellTiter-Glo analysis at the end of the experiments.

### Measurement of intracellular metabolites

The intracellular level of glutamine was determined by ultra-high-performance liquid chromatography-tandem mass spectrometry (UHPLC-MS/MS) with glutamine as the target. A standard solution of L-glutamine was prepared, and calibration curves were drawn. The UHPLC separation was performed using an Agilent 1290 Infinity II series UHPLC system (Agilent Technologies) equipped with a Waters ACQUITY UPLC BEH amide column (100 × 2.1 mm, 1.7 μm). An Agilent 6460 triple quadrupole mass spectrometer (Agilent Technologies) equipped with an AJS electrospray ionization (AJS-ESI) interface was used for assay development. If the concentrations of the metabolites were beyond the linear range, the samples were diluted accordingly to put their concentrations within the range. The final concentration in nmol/L equals the calculated concentration multiplied by the dilution factor.

### Immunofluorescence assay

PANC-1 cells were transfected in slide chambers (NUNC, Denmark) with Flag-LAT2 vector for 24 h. The cells were fixed in methanol, blocked with 10% FBS and then incubated with mouse anti-LAT2 or p-mTOR(Ser2448) antibody and rabbit anti-LDHB antibody. The LAT2 and p-mTOR(Ser2448) staining were detected with an Alexa Fluor 488-labeled goat anti-mouse antibody and the LDHB staining was detected with an Alexa Fluor 647-labeled goat anti-rabbit antibody. DAPI was used for nuclear staining. Images were made using a Leica TCS SP2 microscope.

### Immunoblotting and immunoprecipitation

After transfection for 48 h, total cellular protein was extracted with RIPA lysis buffer (Applygen, Beijing). Total protein (40 μg) was separated on an SDS-PAGE gel and then transferred to a PVDF membrane (Millipore, Billerica, MA). After blocking with 5% nonfat milk at room temperature for 1 h, the membrane was probed with primary antibodies (1:1000, Danvers, MA) overnight at 4 °C. The next day, the membrane was incubated with a horseradish peroxidase-conjugated secondary antibody (1:3000, Applygen, Beijing) at room temperature for 1 h. Proteins were detected by ECL reagents (Millipore, Billerica, MA). For immunoprecipitation, stable PANC-1 transfectants of the pENTER and pENTER LAT2 vectors were seeded in 6-well plates. The cells were then lysed in modified RIPA buffer. The cell lysates were incubated with antibody for 12 h at 4 °C on a rotating plate. The proteins were immunoprecipitated by protein A/G agarose beads (Santa Cruz, USA). The samples were resolved by SDS-PAGE and were subjected to immunoblot analysis.

### Animal experiments

PANC-1 cells stably transfected with LAT2-lentiviral vectors or control lentiviral vectors were injected subcutaneously into the right flank of 6-week-old female BALB/c mice (Shanghai, Chinese Academy of Sciences, China) (5 × 10^6^ cells in 250 μl of PBS per mouse). Each experimental group included five mice. Gemcitabine of 50 mg/kg or equal PBS were administered by intraperitoneal injection two weeks after tumor formation (tumor size between 100 and 200mm^3^), followed by periodic booster shots every three days for two weeks. Tumor size was measured twice a week using a caliper to measure two perpendicular tumor diameters. Tumor volume (mm^3^) was calculated based on the formula: volume (mm^3^) = 1/2 × length × width^2^. Tumor growth inhibition rate was calculated based on the formula: TGI % = (1-(Ti-T0) / (Ci-C0)) × 100. Ti = tumor volume after treatment with GEM, T0 = tumor volume prior to treatment with GEM, Ci = tumor volume after treatment with PBS, C0 = tumor volume prior to treatment with PBS. The tumor-bearing mice were euthanized on the 27th day.

### Statistical analysis

Statistical analysis was performed using SPSS (version 19) and GraphPad PRISM 6 software. Statistical methods included the Wilcoxon signed rank test, Pearson chi-square test, two-tailed Student’s t-test, Kaplan-Meier survival analysis, and Cox proportional hazards regression analysis. All statistical tests included a two-way analysis of variance. Statistical significance was assumed when *P* < 0.05.

## Results

### A high LAT2 level is associated with a poor prognosis

**Fig. 1:**
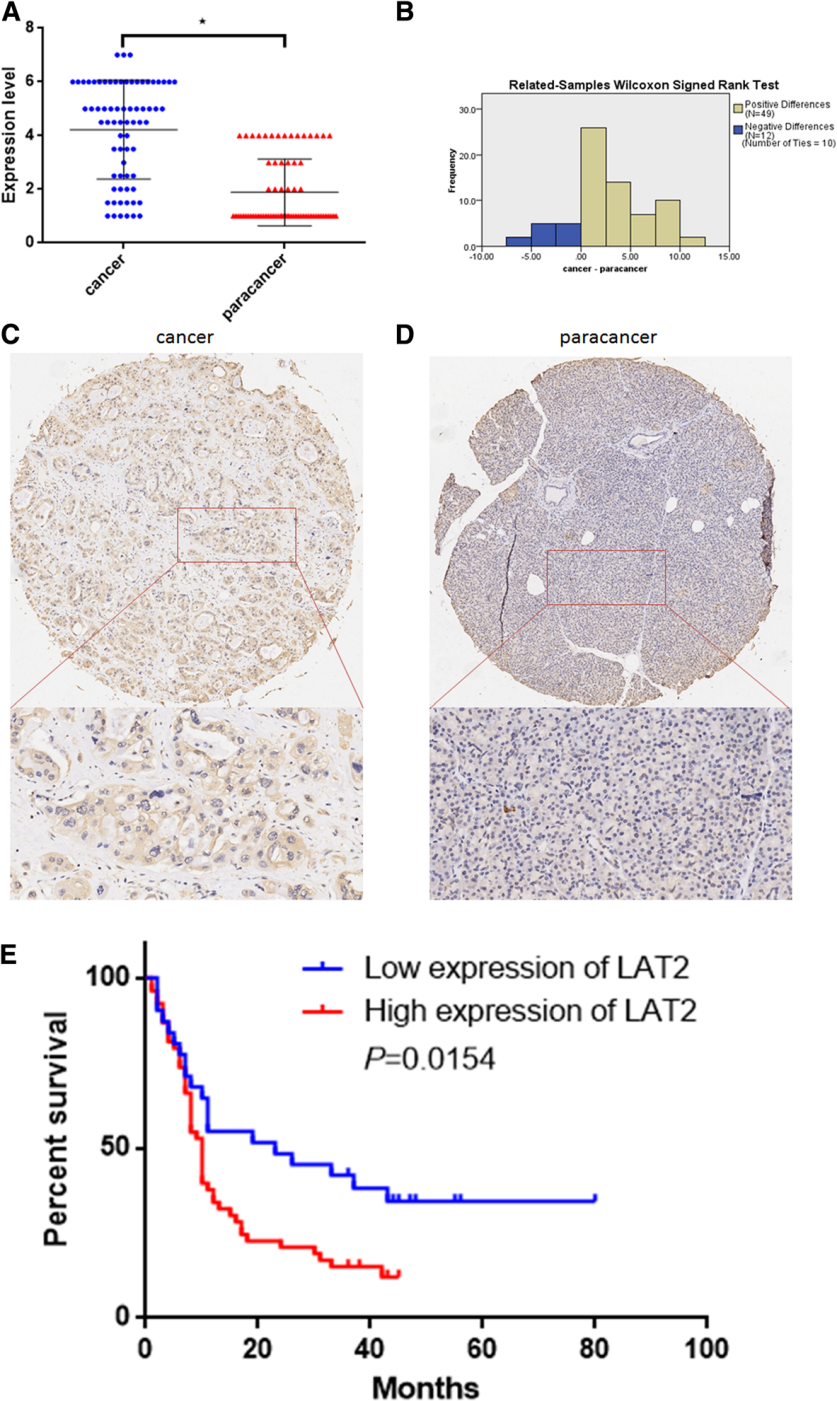
LAT2 is upregulated in pancreatic cancer. **a** The expression level of LAT2 in 71 pancreatic cancer tissue samples was higher than that in the matched paracancerous tissues (*, *P* < 0.05). **b** The Wilcoxon signed rank test was applied to compare the difference in LAT2 expression between the cancer and paracancerous tissues. **c** The expression of LAT2 in pancreatic cancer tissues was evaluated by immunohistochemistry. **d** The expression of LAT2 in pancreatic paracancerous tissues was evaluated by immunohistochemistry. **e** Kaplan-Meier survival analysis indicated that the overall survival time of the high-LAT2-level group is shorter than that of the low-LAT2-level group of patients with pancreatic cancer (*P* = 0.0154)

The expression level of LAT2 in 87 pancreatic cancer tissue samples and 71 matched paracancerous tissues was evaluated by immunohistochemistry (IHC). The Wilcoxon signed rank test was applied to compare the difference in LAT2 expression between the 71 cancer and matched paracancerous tissues. The results revealed that the expression level of LAT2 in the pancreatic cancer tissues was higher than that in the paracancerous tissues (Fig. 1a, b, c, and d). The staining results were scored for high and low LAT2 expression, as depicted in the Materials and Methods section.

The correlation between the LAT2 expression level and the clinicopathological parameters was assessed, and no correlation was shown (see Additional file 1: Table S1). We also explored the correlation between the LAT2 level and the prognosis of pancreatic cancer. The mean follow-up time was 18.63 months (range 1–80 months). Survival analysis indicated that the overall survival rate of patients with a high LAT2 level was lower than that of patients with a low LAT2 level (*P* = 0.0154) (Fig. 1e). Univariate analysis indicated that lymph node staging (*P* = 0.049), differential degree (*P* = 0.002) and LAT2 expression level (*P* = 0.008) were the potential predictive factors for poor prognosis in pancreatic cancer (Table 1). Multivariate analysis demonstrated that a high level of LAT2 was an independent risk factor for poor prognosis in pancreatic cancer (*P* = 0.017) (Table 1).

**Table 1:** Univariate and multivariable analyses of factors predictive of poor overall survival in pancreatic cancer patients

Variables	Univariate analysis			Multivariate analysis		
	Overall survival	95% confidence interval	*P* value	Hazard ratio	95% confidence interval	*P* value
	(Median ± SE,months)					
Sex			0.726			
Male	10 ± 1.063	7.917–12.083				
Female	11 ± 4.433	2.311–19.689				
Age(years old)			0.379			
<65	11 ± 0.980	9.078–12.922				
≥ 65	7 ± 0.833	5.367–8.633				
Locations			0.234			
Head	10 ± 1.440	7.178–12.822				
Body-tail	10 ± 1.571	6.921–13.079				
Perineuronal/vascular/lymphatic invasion			0.280			
No	11 ± 2.60	5.905–16.095				
Yes	10.0 ± 0.957	8.125–11.875				
Tumor staging			0.594			
T1/T2	10.0 ± 1.201	7.646–12.354				
T3/T4	10.0 ± 1.474	7.112–12.888				
Lymph node staging			0.049	2.053	1.228–3.433	0.006
N0	10 ± 3.499	3.141–16.859				
N1/N2	10 ± 1.533	6.996–13.004				
Differential degree			0.002	2.737	1.614–4.644	0.000
High/ moderate	12 ± 2.854	6.406–17.594				
Low	6 ± 1.076	3.890–8.110				
TNM staging			0.614			
I	10 ± 3.177	3.772–16.228				
II/III	10 ± 0.867	8.301–11.699				
LAT2 expression			0.008	1.912	1.122–3.258	0.017
Low	23 ± 9.142	5.081–40.919				
High	9 ± 0.802	7.429–10.571				

### LAT2 decreases gemcitabine sensitivity in vitro and in vivo

**Fig. 2:**
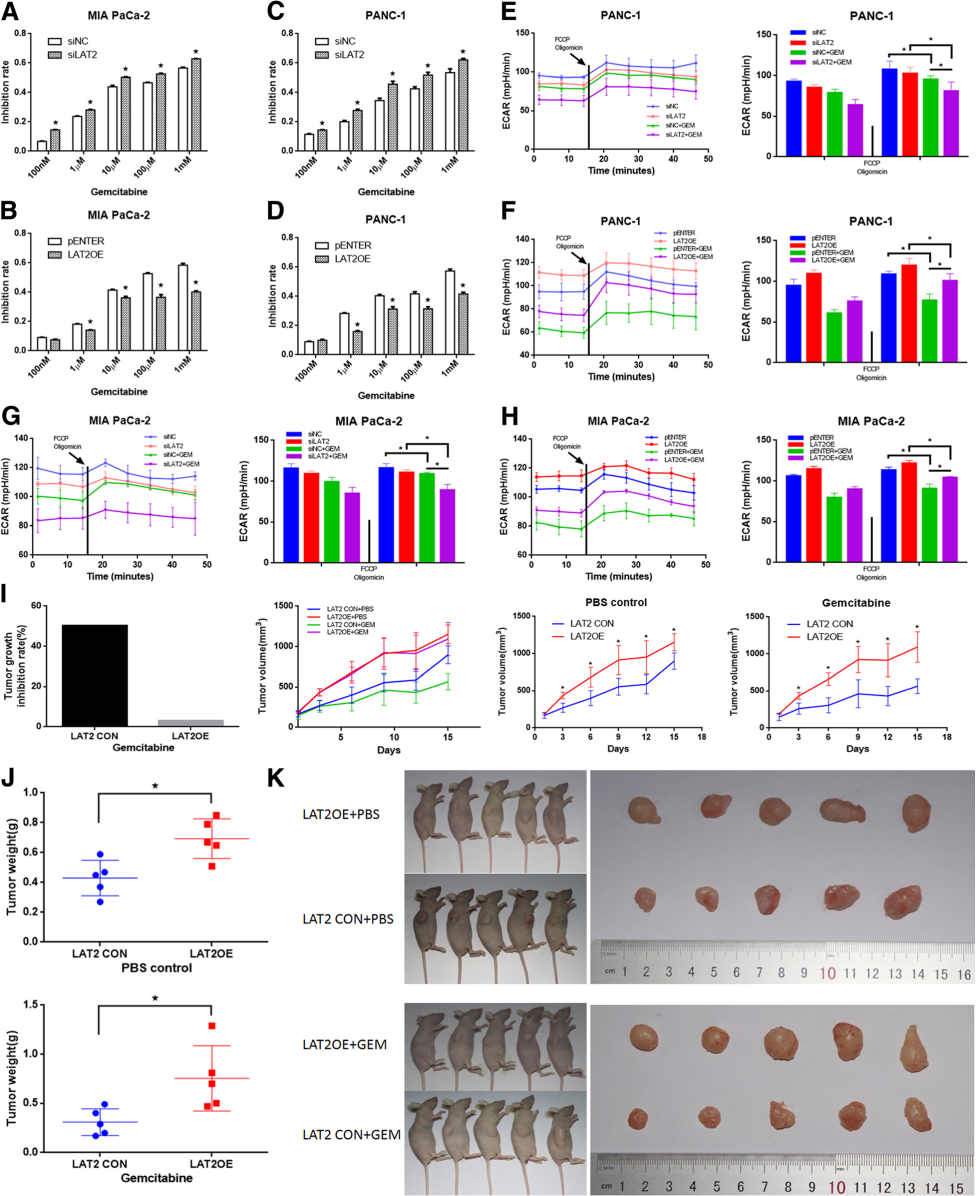
LAT2 decreases gemcitabine sensitivity in vitro and in vivo. **a**, **c** LAT2 knockdown by siLAT2 significantly increased gemcitabine sensitivity in MIA PaCa-2 and PANC-1 cells. **b**, **d** LAT2 overexpression by LAT2 OE plasmids significantly decreased gemcitabine sensitivity in MIA PaCa-2 and PANC-1 cells. **e**, **g** LAT2 knockdown decreased the extracellular acidification rate (ECAR) in MIA PaCa-2 and PANC-1 cells and resulted in an evident reduction in ECAR in MIA PaCa-2 and PANC-1 cells exposed to gemcitabine (10 μM) for 24 h. **f, h** LAT2 overexpression increased ECAR in MIA PaCa-2 and PANC-1 cells and resulted in an evident reduction in ECAR in MIA PaCa-2 and PANC-1 cells exposed to gemcitabine (10 μM) for 24 h. **i** In nude mice treated with gemcitabine or PBS, the tumors generated from pLVX-PANC-1-LAT2 cells grew significantly faster than those generated from the control cells; The tumors generated from cells with a high LAT2 level were significantly larger than those generated from control cells two weeks after treatment with gemcitabine or PBS; The tumor growth inhibition (TGI) rate of the tumors generated from pLVX-PANC-1-LAT2 cells was significantly lower than that of the tumors generated from the control cells. **j** The tumor weight in the pLVX-PANC-1-LAT2 group was significantly heavier than that in the control group. **k** In LAT2 control group, the tumor volume in the GEM treatment group was significantly smaller than that in the PBS control group; In LAT2 OE group, the tumor volume in the GEM treatment group was similar with that in the PBS control group. The data are presented as the mean ± SD. (Student’s t-test; *, *P* < 0.05)

To demonstrate the role of LAT2 in gemcitabine sensitivity in pancreatic cancer, we carried out growth inhibition assays to test the chemosensitivity of pancreatic cancer cells to gemcitabine in vitro and in vivo. As shown in Fig. 2a, b, c, and d, LAT2 knockdown (KD) increased the rate of GEM inhibition in MIA PaCa-2 and PANC-1 cells; in contrast, MIA PaCa-2 and PANC-1 cells with LAT2 overexpression (OE) were less sensitive to GEM than the control cells, which indicated that LAT2 might significantly enhance resistance to GEM in pancreatic cancer cells. Furthermore, we assessed the extracellular acidification rate (ECAR) in LAT2 KD/OE pancreatic cancer cells exposed to GEM and obtained a similar result, which demonstrated that LAT2 could increase ECAR to enhance chemoresistance and that GEM might inhibit the proliferation of pancreatic cancer cells by decreasing ECAR (Fig. 2e, f, g and h). To further determine the effects of LAT2 on chemosensitivity in vivo, we established PANC-1 cell lines that stably overexpressed LAT2 (pLVX-PANC-1-LAT2), subcutaneously injected these cells into nude mice, and then treated the mice with chemotherapy (GEM). The in vivo results revealed that, in mice treated with gemcitabine, the tumors generated from the pLVX-PANC-1-LAT2 cells grew significantly faster than those generated from the control cells (*P* < 0.05) (Fig. 2i). In addition, both the tumor volume and weight in the pLVX-PANC-1-LAT2 group were significantly larger than those in the control group (*P* < 0.05) (Fig. 2i, j, and k). Then, the tumor growth inhibition (TGI) rate was calculated, and the results showed that the TGI% in the pLVX-PANC-1-LAT2 group was significantly lower than that in the control group (Fig. 2k). Taken together, these results suggest that LAT2 decreases the gemcitabine sensitivity of pancreatic cancer cells in vitro and in vivo.

### LAT2 promotes pancreatic cancer cell proliferation in vitro and in vivo

We also assessed the role of LAT2 in proliferation by LAT2 KD/OE in pancreatic cancer cells. As shown in Additional file 2: Figure S1 A-D, LAT2 overexpression significantly promoted proliferation, whereas LAT2 knockdown significantly suppressed proliferation, in MIA PaCa-2 and PANC-1 cells compared with the control cells. In addition, we established the pLVX-PANC-1-LAT2 cell line and subcutaneously injected cells from this line into nude mice. Based on the growth inhibition results of PBS control group, both the tumor volume and weight in the pLVX-PANC-1-LAT2 group were significantly larger than those in the control group (*P* < 0.05) (Fig. 2i, j, and k). As shown in Fig. 2i, the tumors grew significantly faster in the pLVX-PANC-1-LAT2 group than in the control group (*P* < 0.05). In summary, these data indicate that LAT2 promotes pancreatic cancer cell proliferation in vitro and in vivo.

### LAT2 inhibits pancreatic cancer cell apoptosis

To identify the effects of LAT2 on apoptosis in pancreatic cancer cells, we detected the apoptosis rate and apoptosis-related protein changes induced by LAT2 KD/OE in MIA PaCa-2 and PANC-1 cells. As shown in Additional file 2: Figure S2 A-D, siLAT2 significantly increased the percentage of apoptotic MIA PaCa-2 and PANC-1 cells, whereas LAT2 overexpression significantly decreased this percentage compared with the siNC cells. Simultaneously, the results of Western blot analysis revealed that Bax and cleaved caspase-7 were upregulated in MIA PaCa-2 and PANC-1 cells transfected with siLAT2, whereas p21 was downregulated in these cells (Additional file 2: Figure S2 E). In addition, the opposite results were observed in MIA PaCa-2 and PANC-1 cells with LAT2 OE (Additional file 2: Figure S2 E).

### LAT2 activates pancreatic cancer cells glycolysis and alters glutamine metabolism to promote mTOR activation in vitro and in vivo

**Fig. 3:**
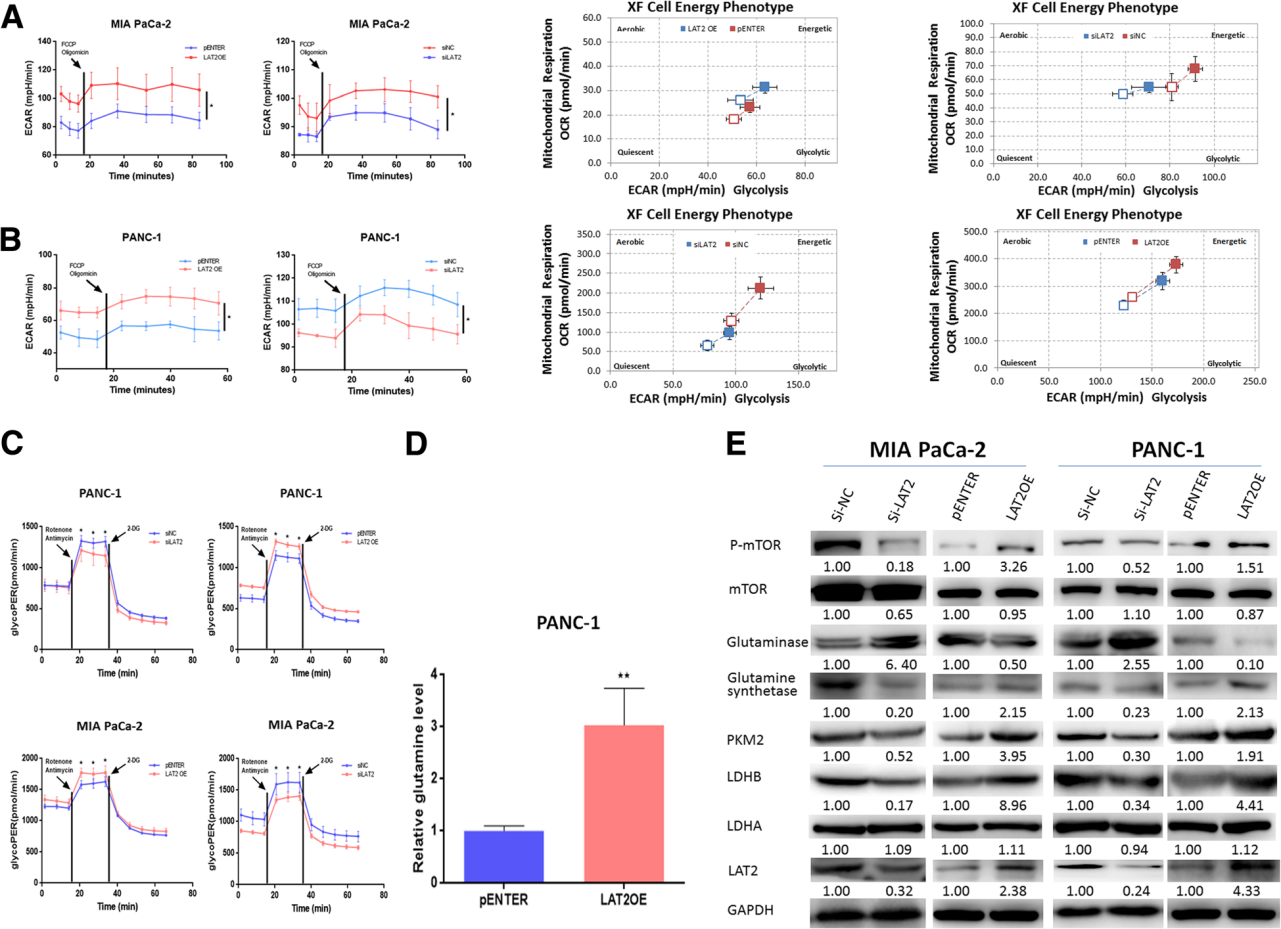
LAT2 activates glycolysis in pancreatic cancer cells and alters glutamine metabolism to activate mTOR in vitro and in vivo*.* **a**, **b** LAT2 knockdown significantly decreased ECAR and OCR in MIA PaCa-2 and PANC-1 cells and promoted the transformation of cell metabolic phenotype from an energetic to a quiescent phenotype; LAT2 overexpression significantly increased ECAR and OCR in MIA PaCa-2 and PANC-1 cells and promoted the transformation of cell metabolic phenotype from a quiescent to an energetic phenotype (P < 0.05). **c** LAT2 knockdown significantly decreased glycoPER in MIA PaCa-2 and PANC-1 cells; LAT2 overexpression significantly increased glycoPER in MIA PaCa-2 and PANC-1 cells (P < 0.05). **d** LAT2 overexpression significantly increased the intracellular level of glutamine in PANC-1 cells. **e** The expression level of mTOR pathway molecules and key enzymes involved in glycolysis and glutamine metabolism was assessed, and the results revealed that LAT2 upregulated LDHB, PKM2, glutamine synthetase and p-mTOR and downregulated glutaminase. Otherwise, LAT2 did not regulate the expression of LDHA. The data are presented as the mean ± SD. (Student’s t-test; **, *P* < 0.01)

We also confirmed the role of LAT2 in glycolysis and glutamine metabolism in pancreatic cancer cells. ECAR, cell energy phenotype and proton efflux rate from glycolysis (glycoPER) were measured with an XF96 Extracellular Flux Analyzer. As shown in Fig. 3a and b, ECAR was increased in MIA PaCa-2 and PANC-1 cells with LAT2 OE and was decreased in cells transfected with siLAT2. We then measured the metabolic phenotype changes, and the results indicated that LAT2 could promote the transformation from a quiescent to a glycolytic or an energetic metabolic phenotype. We also confirmed that LAT2 increased glycoPER in MIA PaCa-2 and PANC-1 cells with LAT2 OE compared with the control cells, and vice versa (Fig. 3c). Then, the intracellular level of glutamine was determined by UHPLC-MS/MS, and the results showed that the intracellular level of glutamine was upregulated in pancreatic cancer cells with LAT2 OE (Fig. 3d). In addition, we detected the protein levels of mTOR pathway and key enzymes involved in the regulation of glycolysis and glutamine metabolism. The results revealed that LAT2 OE increased the levels of LDHB and PKM2, both of which are key molecules involved in promoting glycolysis. In contrast, LAT2 OE could decrease the level of glutaminase and increase the level of glutamine synthetase (Fig. 3e). In vivo, the IHC results from the above-described model of subcutaneously implanted tumors in nude mice revealed that the levels of LDHB, p-mTOR^Ser2448^ and glutamine synthetase were increased in the pLVX-PANC-1-LAT2 group compared with those in the control group; however, the level of glutaminase was decreased (Additional file 2: Figure S3). As shown in Figs. 3e, 6g, and i, both LAT2 and exogenous glutamine activated the mTOR pathway, and mTOR activation (phosphorylation at the Ser2448 site) induced by LAT2 was dependent on glutamine. Glutamine deprivation in MIA PaCa-2 and PANC-1 cells could reverse the mTOR activation induced by LAT2. Thus, all these results show that LAT2 activates glycolysis and increases the intracellular level of glutamine to promote mTOR activation.

### LAT2 binds to p-mTOR^Ser2448^ to upregulate LDHB and activate glycolysis

**Fig. 4:**
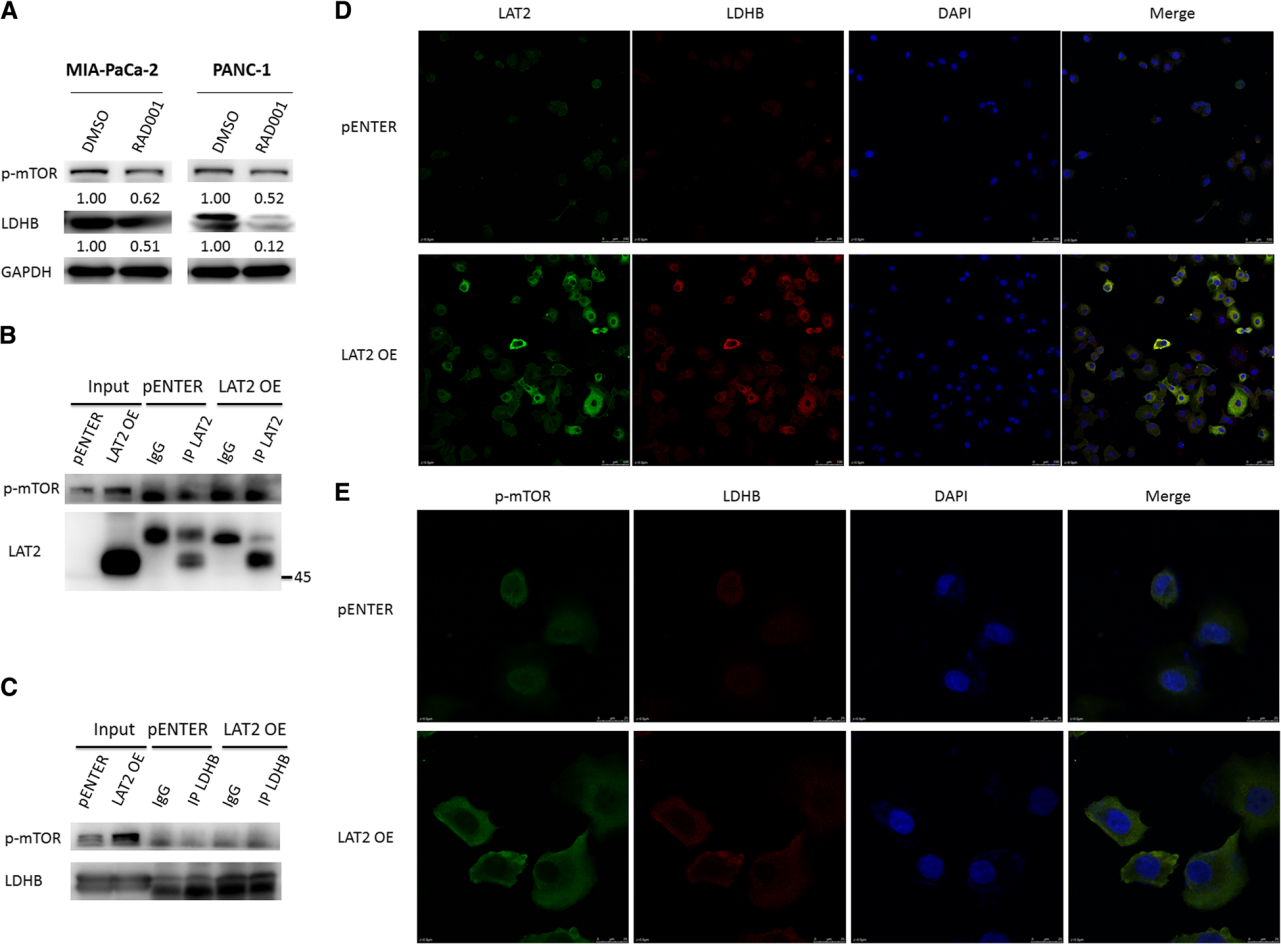
LAT2 binds to p-mTOR^Ser2448^ to upregulate LDHB and activate glycolysis. **a** Western blotting showed that mTOR inhibition (RAD001) downregulated LDHB in MIA PaCa-2 and PANC-1 cells. **b** Immunoprecipitation was used to confirm that LAT2 could bind to p-mTOR^Ser2448^ to activate mTOR. **c** Immunoprecipitation was used to confirm that p-mTOR^Ser2448^ could bind to LDHB to upregulate the expression of LDHB. **d, e** Immunofluorescence was used to confirm that LAT2 could bind to p-mTOR^Ser2448^ to upregulate LDHB; LAT2 was localized to the cytomembrane and cytoplasm; however, both p-mTOR and LDHB were localized to the cytoplasm

To further explore the mechanisms by which LAT2 regulates lactate dehydrogenase B (LDHB) and glycolysis, we performed immunofluorescence and coimmunoprecipitation assays to confirm the relationship between LAT2 and LDHB. As shown in Figs. 3e and 4a, b, c, d and e, LAT2 increased mTOR phosphorylation and upregulated LDHB in MIA PaCa-2 and PANC-1 cells; however, the mTOR inhibitor RAD001 could downregulate LDHB. LAT2 was localized in the cytomembrane and cytoplasm; however, p-mTOR and LDHB were all localized in the cytoplasm. Then, coimmunoprecipitation was used to confirm that LAT2 could bind to p-mTOR^Ser2448^ to activate mTOR and that p-mTOR^Ser2448^ could bind to LDHB to upregulate its expression level (Fig. 4b and c). Glycolysis could be enhanced by LDHB upregulation. Taken together, these data indicate that LAT2 binds to p-mTOR^Ser2448^ to upregulate LDHB and activate glycolysis.

### RAD001 reverses the LAT2-induced decrease in chemosensitivity

**Fig. 5:**
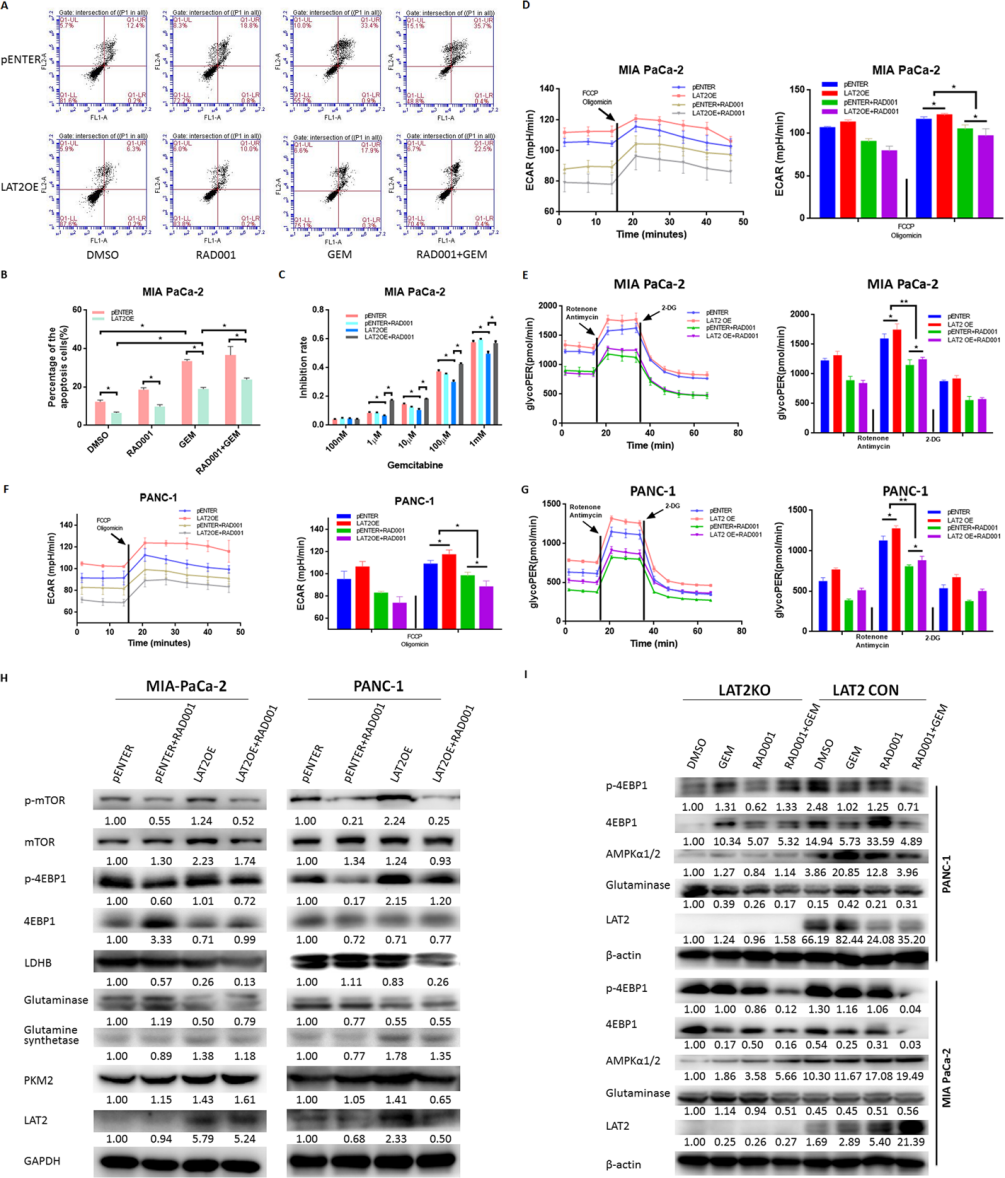
LAT2 activates mTOR to regulate apoptosis, glycolysis and gemcitabine sensitivity in pancreatic cancer cells. **a**, **b** LAT2 suppressed apoptosis in MIA PaCa-2 cells with LAT2 overexpression, which could be reversed by RAD001; gemcitabine induced apoptosis in MIA PaCa-2 cells, and RAD001 reversed the inhibition of apoptosis induced by gemcitabine in MIA PaCa-2 cells with LAT2 overexpression. **c** RAD001 increased gemcitabine sensitivity in MIA PaCa-2 cells with LAT2 overexpression. **d-g** Both ECAR and glycoPER were increased in MIA PaCa-2 and PANC-1 cells with LAT2 overexpression compared with the control cells, and this increase could be reversed by RAD001. **h** RAD001 downregulated the expression of LDHB and glutamine synthetase in MIA PaCa-2 and PANC-1 cells with LAT2 overexpression; RAD001 downregulated LAT2 expression in PANC-1 cells with LAT2 overexpression. **i** LAT2 knockout upregulated glutaminase expression, inhibited the mTOR pathway (thereby downregulating p-4EBP1) and downregulated AMPKα1/2 expression; and gemcitabine suppressed the mTOR pathway in PANC-1 cells dependent on LAT2. The data are presented as the mean ± SD. (Student’s t-test; *, *P* < 0.05)

We also assessed the role of mTOR in the GEM sensitivity of pancreatic cancer cells. LAT2 suppressed apoptosis in pancreatic cancer cells with LAT2 OE; this suppression could be reversed by RAD001 treatment. Otherwise, GEM could induce apoptosis in pancreatic cancer cells, and RAD001 could reverse the inhibition of apoptosis induced by GEM in pancreatic cancer cells with LAT2 OE (Fig. 5a and b). Simultaneously, RAD001 could increase the GEM sensitivity of LAT2 OE pancreatic cancer cells (Fig. 5c). The above results demonstrate that RAD001 reverses the LAT2-induced decrease in chemosensitivity in pancreatic cancer cells.

### RAD001 reverses the LAT2-induced activation of glycolysis

To identify the effects of mTOR on glycolysis in pancreatic cancer cells, we then detected ECAR and glycoPER in MIA PaCa-2 and PANC-1 cells with LAT2 OE that were treated with RAD001. As shown in Fig. 5d, e, f and g, the results revealed that both ECAR and glycoPER were increased in the LAT2 OE pancreatic cancer cells compared with the control cells and that this increase could be reversed by RAD001 treatment. In contrast, RAD001 treatment could downregulate the expression levels of LDHB and glutamine synthetase in MIA PaCa-2 and PANC-1 cells with LAT2 OE (Fig. 5h). LDHB is involved in glycolysis regulation, and glutamine synthetase is involved in glutamine metabolism. Thus, the above data indicate that RAD001 reverses LAT2-induced activation of glycolysis.

### A high LDHB level is associated with a poor prognosis

Then, we detected the expression level of LDHB in 87 pancreatic cancer tissue samples and 71 matched paracancerous tissues using IHC, with two cases of IHC failure. The results indicated that the expression level of LDHB in the cancer tissues was higher than that in the paracancerous tissues (Additional file 2: Figure S4 A-C).

We also investigated the correlation between the LDHB expression level and the prognosis of pancreatic cancer. The survival analysis results revealed that the overall survival time of patients with a high LDHB level was shorter than that of those with a low LDHB level (*P* = 0.0292) (Additional file 2: Figure S4 D). Further analysis indicated that the overall survival time of patients with high levels of LDHB and LAT2 expression was shorter than that of those with other LDHB and LAT2 statuses (*P* = 0.0044) (Additional file 2: Figure S4 E). Thus, these results indicate that high levels of both LDHB and LAT2 are associated with a poor prognosis.

### LAT2 targets glutamine-dependent mTOR activation to regulate apoptosis, glycolysis and chemosensitivity in pancreatic cancer cells

**Fig. 6:**
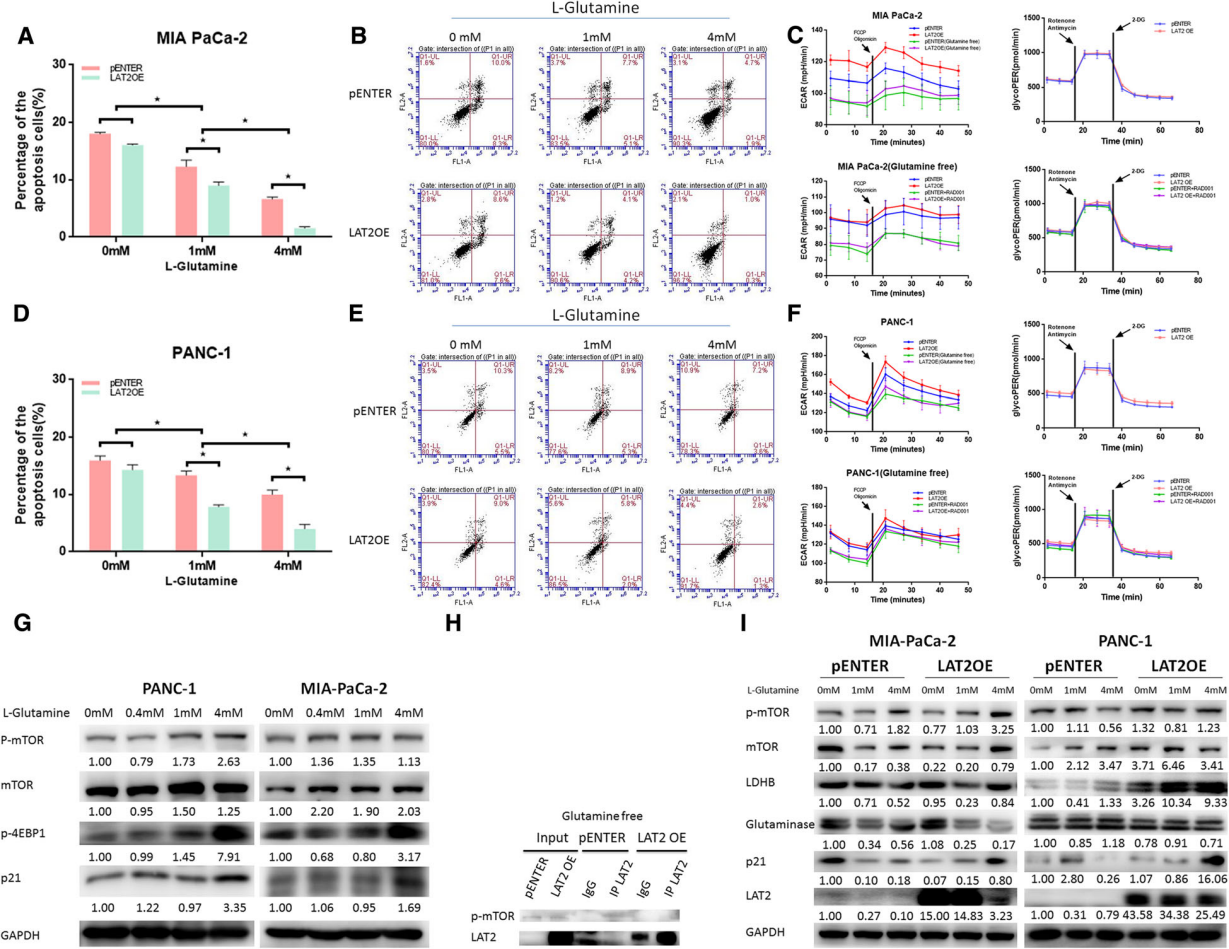
LAT2 targets glutamine-dependent mTOR activation to regulate apoptosis, glycolysis and chemosensitivity in pancreatic cancer cells. **a, b, d, e** The percentage of apoptotic MIA PaCa-2 and PANC-1 cells with LAT2 OE was higher than the percentage of apoptotic control cells when the L-glutamine concentration was normal (4 mM); however, glutamine deprivation reversed the decrease in the percentage of apoptotic cells induced by LAT2; glutamine deprivation increased the percentage of apoptotic MIA PaCa-2 and PANC-1 cells. **c, f** Glutamine deprivation reversed the increase in ECAR and glycoPER induced by LAT2 in MIA PaCa-2 and PANC-1 cells; RAD001 decreased ECAR but not glycoPER in MIA PaCa-2 and PANC-1 cells, regardless of LAT2 expression level. **g** Exogenous L-glutamine activated the mTOR pathway and upregulated p-mTOR, p-4EBP1 and p21 in MIA PaCa-2 and PANC-1 cells. **h** Glutamine deprivation promoted the dissociation of LAT2 and p-mTOR^Ser2448^. **i** Glutamine deprivation in MIA PaCa-2 and PANC-1 cells reversed the mTOR activation induced by LAT2 OE. The data are presented as the mean ± SD. (Student’s t-test; *, *P* < 0.05)

Then, we investigated whether the process by which LAT2 targets mTOR activation to regulate apoptosis, glycolysis and chemosensitivity in pancreatic cancer cells was dependent on glutamine. As shown in Fig. 6a, b, d, and e, the percentage of apoptotic cells in both MIA PaCa-2 and PANC-1 cells with LAT2 OE were higher than that in the control cells when the L-glutamine concentration was normal (4 mM); however, glutamine deprivation (L-glutamine concentration 0 mM) could reverse the LAT-2 induced decrease in the percentage of apoptotic cells. Similarly, glutamine deprivation could reverse the LAT2-induced increase in ECAR and glycoPER in MIA PaCa-2 and PANC-1 cells. Moreover, RAD001 could decrease ECAR but not glycoPER in MIA PaCa-2 and PANC-1 cells regardless of the LAT2 expression level (Fig. 6c and f). Next, we assessed the role of glutamine in the interaction between LAT2 and p-mTOR^Ser2448^. Interestingly, the previous results indicated that LAT2 could bind to p-mTOR^Ser2448^ to upregulate LDHB and activate glycolysis; however, glutamine deprivation promoted the dissociation of LAT2 and p-mTOR^Ser2448^ (Fig. 6h). Taken together, the above results demonstrate that LAT2 targets glutamine-dependent mTOR activation to regulate apoptosis, glycolysis and chemosensitivity in pancreatic cancer cells.

### LAT2 interacts with p-mTOR^Ser2448^ to activate glycolysis, increase intracellular glutamine levels and decrease GEM sensitivity in pancreatic cancer cells

**Fig. 7:**
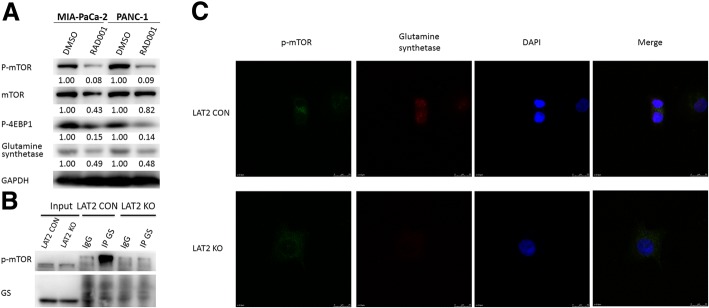
LAT2 binds to p-mTOR^Ser2448^ to upregulate glutamine synthetase and increase the intracellular level of glutamine in pancreatic cancer cells. **a** The Western blotting results showed that RAD001 could downregulate glutamine synthetase in MIA PaCa-2 and PANC-1 cells. **b** LAT2 knockout could inactivate mTOR and decrease the interaction between p-mTOR^Ser2448^ and glutamine synthetase, resulting in a reduced level of glutamine synthetase expression. **c** LAT2 knockout downregulated p-mTOR^Ser2448^ and glutamine synthetase in PANC-1 cells; both p-mTOR^Ser2448^ and glutamine synthetase were localized to the cytoplasm

Next, we explored the mechanisms by which LAT2 regulates glutamine metabolism. As shown in Fig. 7a, b, and c, the results indicated that RAD001 could downregulate glutamine synthetase in MIA PaCa-2 and PANC-1 cells and that the levels of both p-mTOR^Ser2448^ and glutamine synthetase are decreased in pancreatic cancer cells with CRISPR/Cas9-mediated knockout of LAT2 (LAT2 KO). Without LAT2 KO, p-mTOR^Ser2448^ bound to glutamine synthetase to upregulate the expression level of glutamine synthetase. Thus, the above data and previous data indicated that LAT2 could bind to p-mTOR^Ser2448^ to upregulate glutamine synthetase expression, which could increase the intracellular level of glutamine in pancreatic cancer cells. Next, we further assessed the interaction between LAT2 and p-mTOR^Ser2448^. As shown in Fig. 5h, the results revealed that RAD001 treatment could conversely downregulate LAT2 expression in PANC-1 cells with LAT2 OE. LAT2 KO could also upregulate glutaminase expression, inhibit the mTOR pathway (thereby downregulating p-4EBP1) and downregulate AMPKα1/2 expression. In contrast, GEM could inhibit the mTOR pathway in PANC-1 cells dependent on LAT2 (Fig. 5i). Combined with the previous results, these results demonstrated that LAT2 could increase the intracellular level of glutamine and bind to p-mTOR^Ser2448^ to upregulate the levels of LDHB and glutamine synthetase. Then, the upregulated LDHB could activate glycolysis and decrease GEM sensitivity in pancreatic cancer, and the increased glutamine synthetase and decreased glutaminase could further increase the level of intracellular glutamine, which is indispensable for mTOR activation. However, RAD001 treatment could also inhibit LAT2. Consequently, LAT2 interacts with p-mTOR^Ser2448^ to activate glycolysis, increase the intracellular level of glutamine and decrease the GEM sensitivity of pancreatic cancer cells.

## Discussion

In humans, the specific role of LAT2 in pancreatic cancer has not been reported and is still elusive. Our previous study has demonstrated that the expression level of LAT2 in AsPC-1-GEM cells is higher than that in the parental cells, which might indicate that LAT2 plays an important role in chemoresistance in pancreatic cancer. Chemoresistance is regarded as one of the primary causes of poor prognosis and high mortality in pancreatic cancer. In this study, we identified LAT2 as a novel oncogenic protein in pancreatic cancer. We found that the expression levels of both LAT2 and LDHB in pancreatic cancer tissues were higher than those in the paracancerous tissues and that high levels of both LAT2 and LDHB were associated with poor prognosis. In addition, LAT2 could decrease GEM sensitivity, promote proliferation, inhibit apoptosis and activate glycolysis in pancreatic cancer cells, and these effects could be reversed by RAD001 treatment. Mechanistically, LAT2 could regulate the glutamine/p-mTOR^Ser2448^/glutamine synthetase feedback loop to keep mTOR activated and to upregulate LDHB in order to activate glycolysis, thereby promoting chemoresistance in pancreatic cancer cells. We also demonstrated that the decreased apoptosis and increased ECAR and glycoPER induced by the LAT2-mTOR-LDHB pathway in pancreatic cancer cells were dependent on glutamine. Consequently, our data indicate that the LAT2-mTOR-LDHB pathway might be a valuable prognostic predictor and promising therapeutic target in pancreatic cancer.

LAT2 has been reported to play different roles in multiple tumor types. Barollo et al. confirmed that LAT2 was overexpressed in neuroendocrine tumors, including pheochromocytoma and medullary thyroid carcinoma, compared with normal tissues and that it was responsible for dihydroxyphenylalanine uptake [11]. Luo et al. found that LAT2 might play important roles in the proliferation of uterine leiomyoma cells [12]. In breast cancer, SLC7A8 mRNA expression was elevated in samples from estrogen receptor alpha-positive breast cancer patients. However, a high level of SLC7A8 mRNA was significantly associated with longer relapse-free survival in estrogen receptor alpha-positive and lymph node-positive breast cancer [13]. Our study demonstrated that a high LAT2 level was associated with poor overall survival in pancreatic cancer. In terms of chemosensitivity, Rumiato et al. found that single nucleotide polymorphisms mapping to the SLC7A8 gene, in combination with clinical variables, could contribute to the accuracy of the predicted response to platinum chemotherapy in esophageal cancer patients [14]. Meanwhile, another study showed that the expression of the SLC7A8 gene was significantly decreased in the paclitaxel-resistant W1 human ovarian cancer cell line compared with W1 parental cells; thus, SLC7A8 might be involved in reversing drug resistance in ovarian cancer [15]. However, in our previous study, LAT2 showed a higher expression level in AsPC-1-GEM cells than in the parental cells. In this study, we also demonstrated that LAT2 played an oncogenic role in pancreatic cancer and could decrease GEM sensitivity by regulating the glutamine-dependent LAT2-mTOR-LDHB pathway.

Glutamine addiction is a feature of many cancer types. Glutamine, a major source of carbon and nitrogen for sustaining the proliferation of cancer cells, has been reported to be critical for proliferation in pancreatic cancer [16–18]. Otherwise, oncogenic alterations in genes such as MYC and KRAS could reprogram glutamine metabolism in cancer cells [8]. For example, c-MYC has been demonstrated to bind to the promoter regions of high-affinity glutamine importers, thereby causing increased glutamine uptake, and oncogenic KRAS could upregulate enzymes involved in glutamine metabolism [19, 20]. Jewell et al. have demonstrated that glutamine could activate mTORC1, which is important for the growth of both normal and tumor cells [7]. Recent studies have indicated that transporters act as amino acid sensors involved in controlling mTOR recruitment and activation at the surface of multiple intracellular compartments. We have demonstrated that LAT2 could bind to p-mTOR to regulate the glutamine/p-mTOR^ser2448^/glutamine synthetase feedback loop, resulting in an increased intracellular level of glutamine and the sustained activation of mTOR. In contrast, LAT2 could downregulate glutaminase to further increase the intracellular level of glutamine. Activated mTOR could bind to and upregulate LDHB to increase glycolysis and decrease chemosensitivity. Glutamine deprivation could then cause mTOR activation failure and decreased glycolysis. We also demonstrated that RAD001 treatment could downregulate LAT2, which indicated that mTOR activation might contribute to maintaining LAT2 stability. Therefore, LAT2 could bind to p-mTOR^Ser2448^, and they could interact with each other to form a positive feedback loop. Next, p-mTOR^Ser2448^ could bind to and upregulate glutamine synthetase to increase endogenous glutamine levels, thereby forming another positive feedback loop (glutamine/p-mTOR^ser2448^/glutamine synthetase). Meanwhile, mTOR activation depends on exogenous glutamine. Therefore, both positive feedback loops are dependent on exogenous glutamine.

RAD001, a mTOR inhibitor, has been approved for the treatment of pancreatic cancer; however, treatment with single mTOR inhibitors can lead only to disease stabilization rather than regression because mTOR contributes to cellular proliferation rather than cell survival [21]. In this study, we have demonstrated that RAD001 treatment could reverse the decrease in GEM sensitivity caused by LAT2 overexpression in pancreatic cancer. These results indicate that GEM combined with RAD001 might improve chemosensitivity in pancreatic cancer patients with LAT2 overexpression.

In terms of the relationship between mTOR and LDHB, Zha et al. demonstrated that LDHB, which is critical for oncogenic mTOR-mediated tumorigenesis, is a downstream target of mTOR [22]. Our study also confirmed that LAT2 could target mTOR activation to regulate LDHB, which is critical for the conversion of pyruvate to lactate. Then, we assessed the role of the LAT2-mTOR-LDHB pathway in glycolysis, and the results indicated that both LAT2 KD and mTOR inhibition could downregulate LDHB, decrease glycoPER and inhibit glycolysis. In contrast, increased glycoPER has been reported to be associated with decreased chemosensitivity. In addition, we have demonstrated that RAD001 treatment could reverse the decrease in GEM sensitivity induced by LAT2 OE. Therefore, we speculated that LAT2 could regulate the above two positive feedback loops to upregulate LDHB and activate glycolysis, which would promote chemoresistance in pancreatic cancer. However, Cui et al. found that LDHB might act as a suppressor of glycolysis and as a tumor suppressor gene in pancreatic cancer due to promoter hypermethylation [23]. Conversely, a recent study indicated that positive LDHB protein expression was associated with progression and poor prognosis in patients with pancreatic cancer [24]. Otherwise, LDHB has been reported to be an oncogenic protein in multiple tumor types, including uterine cancer, ovarian cancer, colon cancer and breast cancer [25–27]. In this study, we demonstrated that a high LDHB level is associated with poor prognosis in pancreatic cancer.

## Conclusion

In conclusion, we have demonstrated that LAT2 could regulate two glutamine-dependent positive feedback loops (the LAT2/p-mTOR^Ser2448^ loop and the glutamine/p-mTOR^Ser2448^/glutamine synthetase loop) to promote glycolysis and decrease GEM sensitivity in pancreatic cancer. In addition, high levels of both LAT2 and LDHB are poor prognostic predictors in pancreatic cancer. Finally, GEM combined with RAD001 could increase GEM sensitivity in pancreatic cancer patients with LAT2 OE. Therefore, the LAT2-mTOR-LDHB pathway might be a promising therapeutic target in pancreatic cancer, and further study should be conducted to investigate the specific mechanisms between the two positive feedback loops.

## Additional files

Additional file 1: **Table S1.** Correlations of LAT2 level in tissues and clinicopathological parameters. (DOC 44 kb)

Additional file 2: **Figure S1.** LAT2 promotes pancreatic cancer cell proliferation. (A, C) Overexpression of LAT2 in MIA PaCa-2 and PANC-1 cells promoted cell proliferation. (B, D) LAT2 knockdown suppressed cell proliferation. (*, *P* < 0.05). **Figure S2.** LAT2 inhibits pancreatic cancer cell apoptosis. (A, C) LAT2 knockdown in MIA PaCa-2 and PANC-1 cells increased the percentage of apoptotic cells compared with the control. (B, D) Overexpression of LAT2 decreased the percentage of apoptotic cells compared with the control. (E) The Western blotting results revealed that Bax and cleaved caspase-7 were upregulated in MIA PaCa-2 and PANC-1 cells transfected with siLAT2, whereas p21 was downregulated; contrary results were achieved when the cells were transfected with LAT2 OE plasmids. (*, *P* < 0.05). **Figure S3.** LAT2 activates glycolysis and alters glutamine metabolism in vivo. The IHC results revealed that the levels of LAT2, LDHB, p-mTOR^Ser2448^ and glutamine synthetase were increased in tumors generated from pLVX-PANC-1-LAT2 cells in nude mice compared with those in tumors generated from the control cells, whereas the level of glutaminase was decreased. **Figure S4.** A high LDHB level is associated with poor prognosis in pancreatic cancer. (A) The Wilcoxon signed rank test was applied to compare the difference in LDHB expression between cancer and paracancerous tissues. (B, C) The expression level of LDHB in 69 pancreatic cancer tissue samples was higher than that in the matched paracancerous tissues (*, *P* < 0.05). (D, E) Kaplan-Meier survival analysis indicated that the overall survival rate of the high-LDHB and high-LAT2 level group is poorer than that of pancreatic cancer patients with other LDHB and LAT2 statuses (*P* = 0.0044). **Figure S5.** LAT2 regulates glutamine-dependent mTOR activation to promote glycolysis and decrease gemcitabine sensitivity. This diagram shows the mechanisms by which LAT2 regulates glycolysis and gemcitabine sensitivity in pancreatic cancer. ASCT2, ASC family transporter 2 (also called SLC1A5). (ZIP 7755 kb)

## Abbreviations

AJCC: American Joint Committee on Cancer
ECAR: Extracellular acidification rate
GEM: Gemcitabine
glycoPER: Proton efflux rate from glycolysis
IHC: Immunohistochemistry
KD: Knockdown
LAT2 KO: Knockout of LAT2
LAT2: L-type amino acid transporter 2
LDHB: Lactate dehydrogenase B
mTOR: Mechanistic target of rapamycin
NEAAs: Nonessential amino acids
OD: Optical density
OE: Overexpression
TCA: Tricarboxylic acid cycle
UHPLC-MS/MS: Ultra-high-performance liquid chromatography- tandem mass spectrometry
