# Supplementary material for: LAT2 regulates glutamine-dependent mTOR activation to promote glycolysis and chemoresistance in pancreatic cancer
Source: J Exp Clin Cancer Res. 2018 Nov 12;37:274. doi: 10.1186/s13046-018-0947-4 (PMC6233565; doi:10.1186/s13046-018-0947-4)
Supplement: Supplementary file 1 — Table S1. Correlations of LAT2 level in tissues and clinicopathological parameters. (DOC 44 kb) [file 13046_2018_947_MOESM1_ESM.doc]

### Additional file 1

Table S1 Correlations of LAT2 level in tissues and clinicopathological parameters

| **Variables** | **LAT2 expression** | | |
| --- | --- | --- | --- |
|  | **Low level group**  **（n=31）** | **High level group**  **（n=56）** | ***P* value** |
| **Gender** |  |  |  |
| Male | 21 | 35 | 0.625 |
| Female | 10 | 21 |  |
| **Age^*^** |  |  |  |
| ＜65 | 21 | 31 | 0.186 |
| ≥65 | 9 | 25 |  |
| **Location** |  |  |  |
| Head | 24 | 35 | 0.154 |
| Body-tail | 7 | 21 |  |
| **Pathological grading** |  |  |  |
| Ⅰ/Ⅱ | 19 | 33 | 0.874 |
| Ⅲ/Ⅳ | 12 | 23 |  |
| **Tumor staging** |  |  |  |
| T1/T2 | 42 | 19 | 0.83 |
| T3/T4 | 23 | 15 |  |
| **Lymph node staging** |  |  |  |
| N0 | 19 | 30 | 0.487 |
| N1/N2 | 12 | 26 |  |
| **TNM staging** |  |  |  |
| Ⅰ | 9 | 17 | 0.897 |
| Ⅱ/Ⅲ | 22 | 39 |  |
| **Perineuronal/vascular/lymphatic invasion** |  |  |  |
| No | 20 | 33 | 0.609 |
| Yes | 11 | 23 |  |

***: One patient’s age information was lost.**
