# Supplementary figures and images for: LAT2 regulates glutamine-dependent mTOR activation to promote glycolysis and chemoresistance in pancreatic cancer
Source: J Exp Clin Cancer Res. 2018 Nov 12;37:274. doi: 10.1186/s13046-018-0947-4 (PMC6233565; doi:10.1186/s13046-018-0947-4)

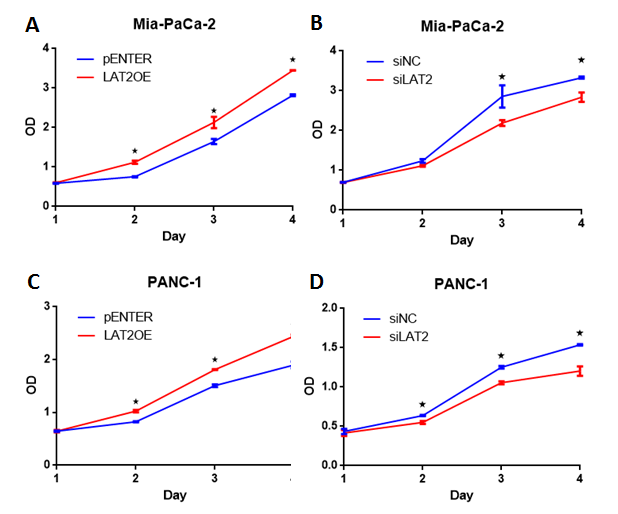

Supplement: Supplementary file 2 — Figure S1. LAT2 promotes pancreatic cancer cell proliferation. (A, C) Overexpression of LAT2 in MIA PaCa-2 and PANC-1 cells promoted cell proliferation. (B, D) LAT2 knockdown suppressed cell proliferation. (*, P < 0.05). Figure S2. LAT2 inhibits pancreatic cancer cell apoptosis. (A, C) LAT2 knockdown in MIA PaCa-2 and PANC-1 cells increased the percentage of apoptotic cells compared with the control. (B, D) Overexpression of LAT2 decreased the percentage of apoptotic cells compared with the control. (E) The Western blotting results revealed that Bax and cleaved caspase-7 were upregulated in MIA PaCa-2 and PANC-1 cells transfected with siLAT2, whereas p21 was downregulated; contrary results were achieved when the cells were transfected with LAT2 OE plasmids. (*, P < 0.05). Figure S3. LAT2 activates glycolysis and alters glutamine metabolism in vivo. The IHC results revealed that the levels of LAT2, LDHB, p-mTORSer2448 and glutamine synthetase were increased in tumors generated from pLVX-PANC-1-LAT2 cells in nude mice compared with those in tumors generated from the control cells, whereas the level of glutaminase was decreased. Figure S4. A high LDHB level is associated with poor prognosis in pancreatic cancer. (A) The Wilcoxon signed rank test was applied to compare the difference in LDHB expression between cancer and paracancerous tissues. (B, C) The expression level of LDHB in 69 pancreatic cancer tissue samples was higher than that in the matched paracancerous tissues (*, P < 0.05). (D, E) Kaplan-Meier survival analysis indicated that the overall survival rate of the high-LDHB and high-LAT2 level group is poorer than that of pancreatic cancer patients with other LDHB and LAT2 statuses (P = 0.0044). Figure S5. LAT2 regulates glutamine-dependent mTOR activation to promote glycolysis and decrease gemcitabine sensitivity. This diagram shows the mechanisms by which LAT2 regulates glycolysis and gemcitabine sensitivity in pancreatic cancer. ASCT2, ASC family transporter [file 13046_2018_947_MOESM2_ESM.zip › Figure S1.tif]

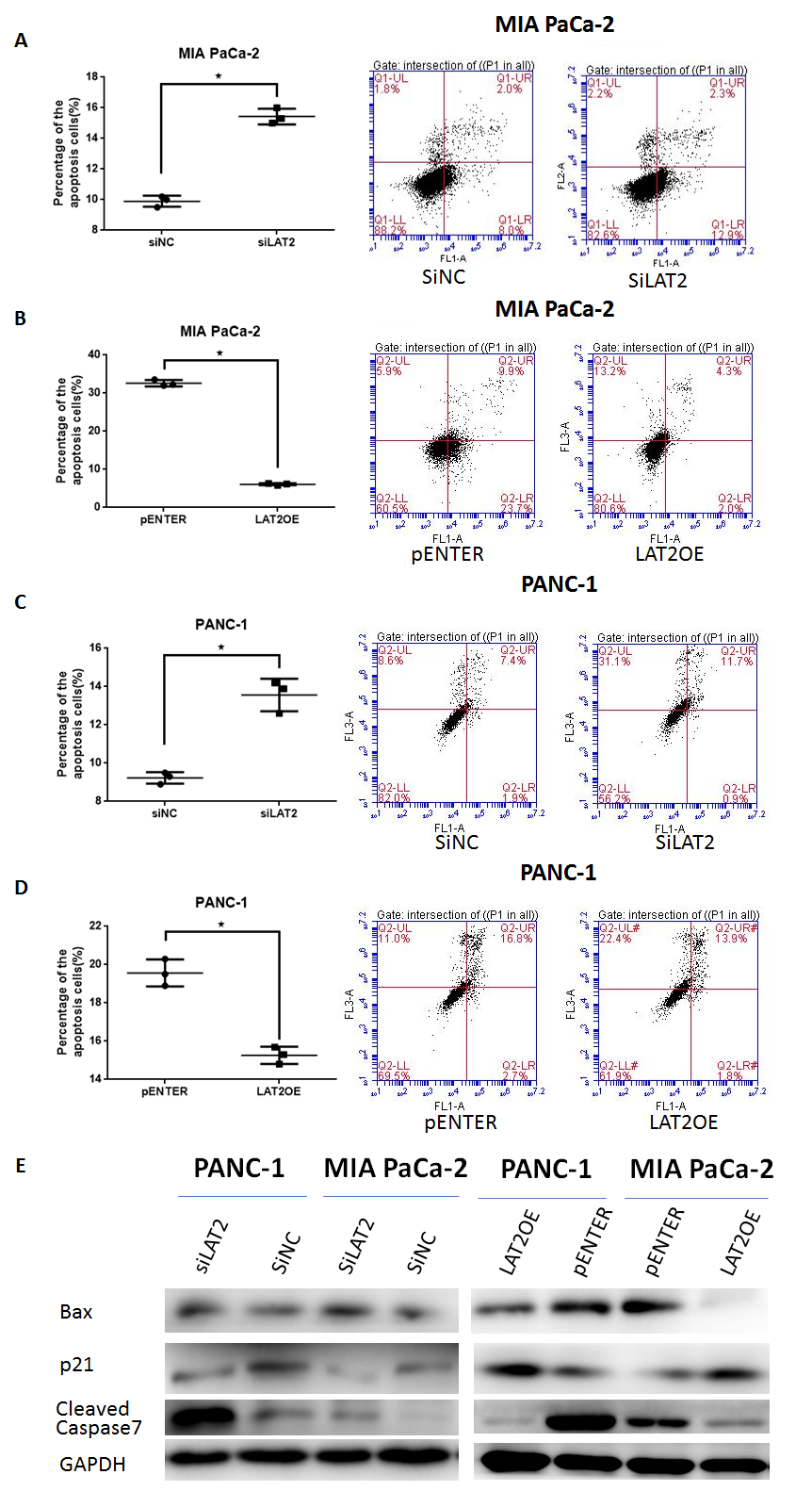

Supplement: Supplementary file 2 — Figure S1. LAT2 promotes pancreatic cancer cell proliferation. (A, C) Overexpression of LAT2 in MIA PaCa-2 and PANC-1 cells promoted cell proliferation. (B, D) LAT2 knockdown suppressed cell proliferation. (*, P < 0.05). Figure S2. LAT2 inhibits pancreatic cancer cell apoptosis. (A, C) LAT2 knockdown in MIA PaCa-2 and PANC-1 cells increased the percentage of apoptotic cells compared with the control. (B, D) Overexpression of LAT2 decreased the percentage of apoptotic cells compared with the control. (E) The Western blotting results revealed that Bax and cleaved caspase-7 were upregulated in MIA PaCa-2 and PANC-1 cells transfected with siLAT2, whereas p21 was downregulated; contrary results were achieved when the cells were transfected with LAT2 OE plasmids. (*, P < 0.05). Figure S3. LAT2 activates glycolysis and alters glutamine metabolism in vivo. The IHC results revealed that the levels of LAT2, LDHB, p-mTORSer2448 and glutamine synthetase were increased in tumors generated from pLVX-PANC-1-LAT2 cells in nude mice compared with those in tumors generated from the control cells, whereas the level of glutaminase was decreased. Figure S4. A high LDHB level is associated with poor prognosis in pancreatic cancer. (A) The Wilcoxon signed rank test was applied to compare the difference in LDHB expression between cancer and paracancerous tissues. (B, C) The expression level of LDHB in 69 pancreatic cancer tissue samples was higher than that in the matched paracancerous tissues (*, P < 0.05). (D, E) Kaplan-Meier survival analysis indicated that the overall survival rate of the high-LDHB and high-LAT2 level group is poorer than that of pancreatic cancer patients with other LDHB and LAT2 statuses (P = 0.0044). Figure S5. LAT2 regulates glutamine-dependent mTOR activation to promote glycolysis and decrease gemcitabine sensitivity. This diagram shows the mechanisms by which LAT2 regulates glycolysis and gemcitabine sensitivity in pancreatic cancer. ASCT2, ASC family transporter [file 13046_2018_947_MOESM2_ESM.zip › Figure S2.tif]

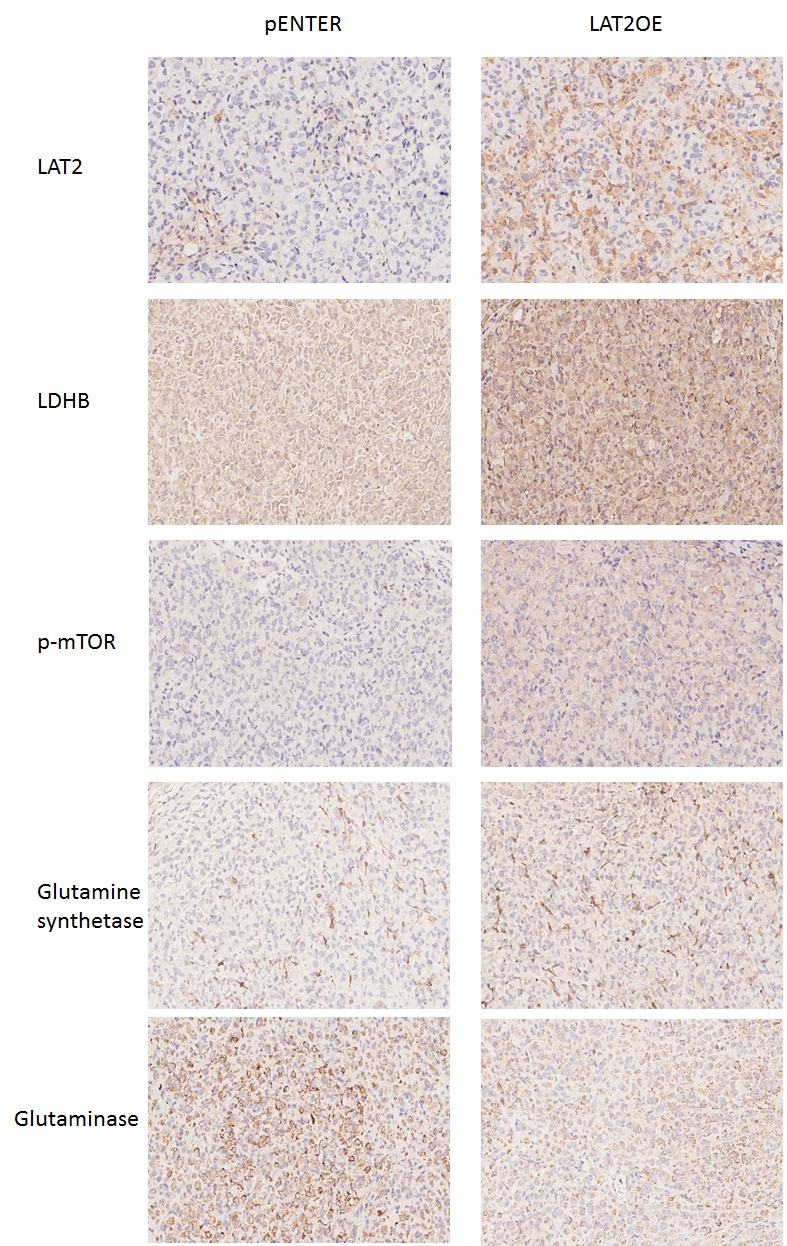

Supplement: Supplementary file 2 — Figure S1. LAT2 promotes pancreatic cancer cell proliferation. (A, C) Overexpression of LAT2 in MIA PaCa-2 and PANC-1 cells promoted cell proliferation. (B, D) LAT2 knockdown suppressed cell proliferation. (*, P < 0.05). Figure S2. LAT2 inhibits pancreatic cancer cell apoptosis. (A, C) LAT2 knockdown in MIA PaCa-2 and PANC-1 cells increased the percentage of apoptotic cells compared with the control. (B, D) Overexpression of LAT2 decreased the percentage of apoptotic cells compared with the control. (E) The Western blotting results revealed that Bax and cleaved caspase-7 were upregulated in MIA PaCa-2 and PANC-1 cells transfected with siLAT2, whereas p21 was downregulated; contrary results were achieved when the cells were transfected with LAT2 OE plasmids. (*, P < 0.05). Figure S3. LAT2 activates glycolysis and alters glutamine metabolism in vivo. The IHC results revealed that the levels of LAT2, LDHB, p-mTORSer2448 and glutamine synthetase were increased in tumors generated from pLVX-PANC-1-LAT2 cells in nude mice compared with those in tumors generated from the control cells, whereas the level of glutaminase was decreased. Figure S4. A high LDHB level is associated with poor prognosis in pancreatic cancer. (A) The Wilcoxon signed rank test was applied to compare the difference in LDHB expression between cancer and paracancerous tissues. (B, C) The expression level of LDHB in 69 pancreatic cancer tissue samples was higher than that in the matched paracancerous tissues (*, P < 0.05). (D, E) Kaplan-Meier survival analysis indicated that the overall survival rate of the high-LDHB and high-LAT2 level group is poorer than that of pancreatic cancer patients with other LDHB and LAT2 statuses (P = 0.0044). Figure S5. LAT2 regulates glutamine-dependent mTOR activation to promote glycolysis and decrease gemcitabine sensitivity. This diagram shows the mechanisms by which LAT2 regulates glycolysis and gemcitabine sensitivity in pancreatic cancer. ASCT2, ASC family transporter [file 13046_2018_947_MOESM2_ESM.zip › Figure S3.tif]

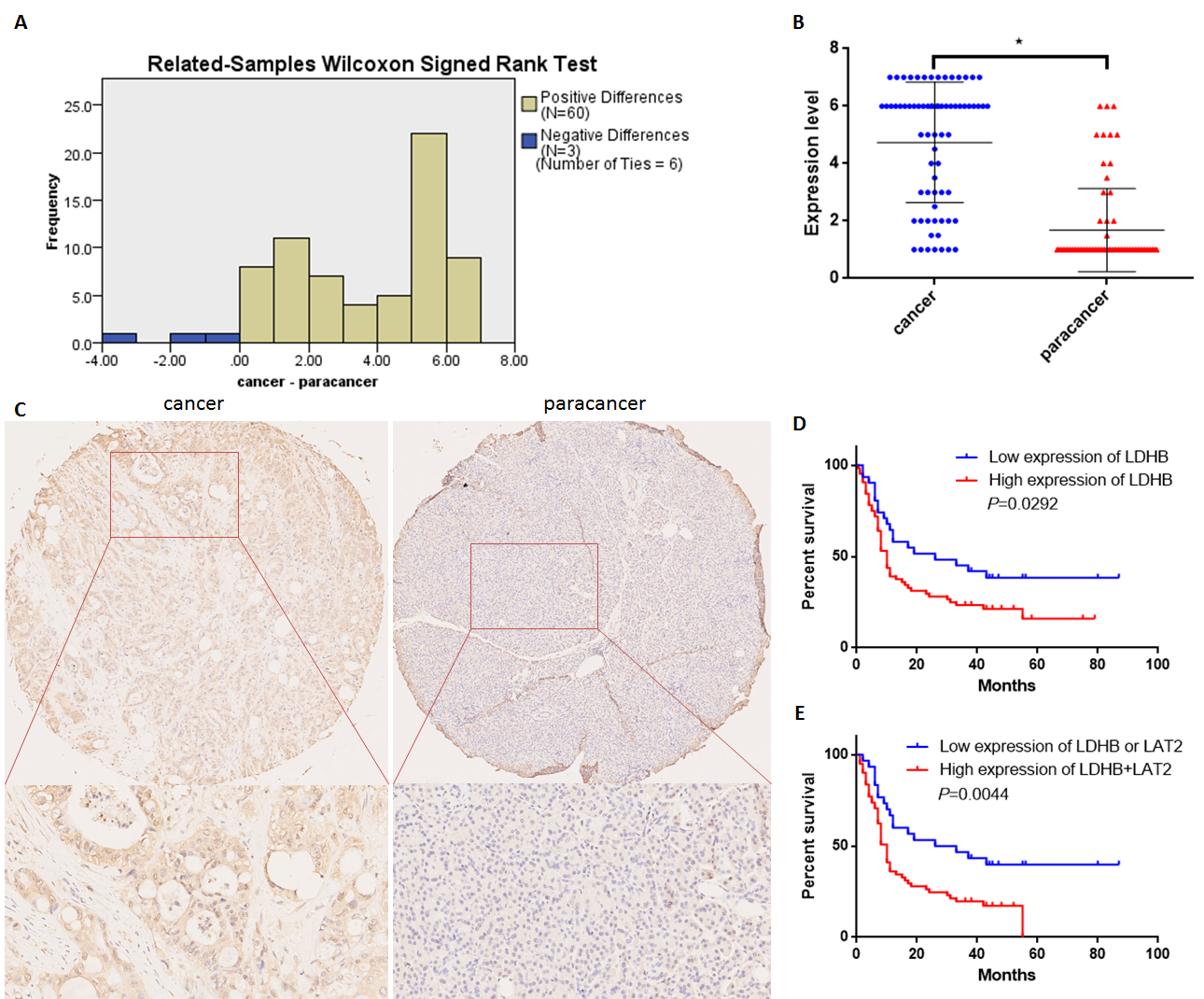

Supplement: Supplementary file 2 — Figure S1. LAT2 promotes pancreatic cancer cell proliferation. (A, C) Overexpression of LAT2 in MIA PaCa-2 and PANC-1 cells promoted cell proliferation. (B, D) LAT2 knockdown suppressed cell proliferation. (*, P < 0.05). Figure S2. LAT2 inhibits pancreatic cancer cell apoptosis. (A, C) LAT2 knockdown in MIA PaCa-2 and PANC-1 cells increased the percentage of apoptotic cells compared with the control. (B, D) Overexpression of LAT2 decreased the percentage of apoptotic cells compared with the control. (E) The Western blotting results revealed that Bax and cleaved caspase-7 were upregulated in MIA PaCa-2 and PANC-1 cells transfected with siLAT2, whereas p21 was downregulated; contrary results were achieved when the cells were transfected with LAT2 OE plasmids. (*, P < 0.05). Figure S3. LAT2 activates glycolysis and alters glutamine metabolism in vivo. The IHC results revealed that the levels of LAT2, LDHB, p-mTORSer2448 and glutamine synthetase were increased in tumors generated from pLVX-PANC-1-LAT2 cells in nude mice compared with those in tumors generated from the control cells, whereas the level of glutaminase was decreased. Figure S4. A high LDHB level is associated with poor prognosis in pancreatic cancer. (A) The Wilcoxon signed rank test was applied to compare the difference in LDHB expression between cancer and paracancerous tissues. (B, C) The expression level of LDHB in 69 pancreatic cancer tissue samples was higher than that in the matched paracancerous tissues (*, P < 0.05). (D, E) Kaplan-Meier survival analysis indicated that the overall survival rate of the high-LDHB and high-LAT2 level group is poorer than that of pancreatic cancer patients with other LDHB and LAT2 statuses (P = 0.0044). Figure S5. LAT2 regulates glutamine-dependent mTOR activation to promote glycolysis and decrease gemcitabine sensitivity. This diagram shows the mechanisms by which LAT2 regulates glycolysis and gemcitabine sensitivity in pancreatic cancer. ASCT2, ASC family transporter [file 13046_2018_947_MOESM2_ESM.zip › Figure S4.tif]

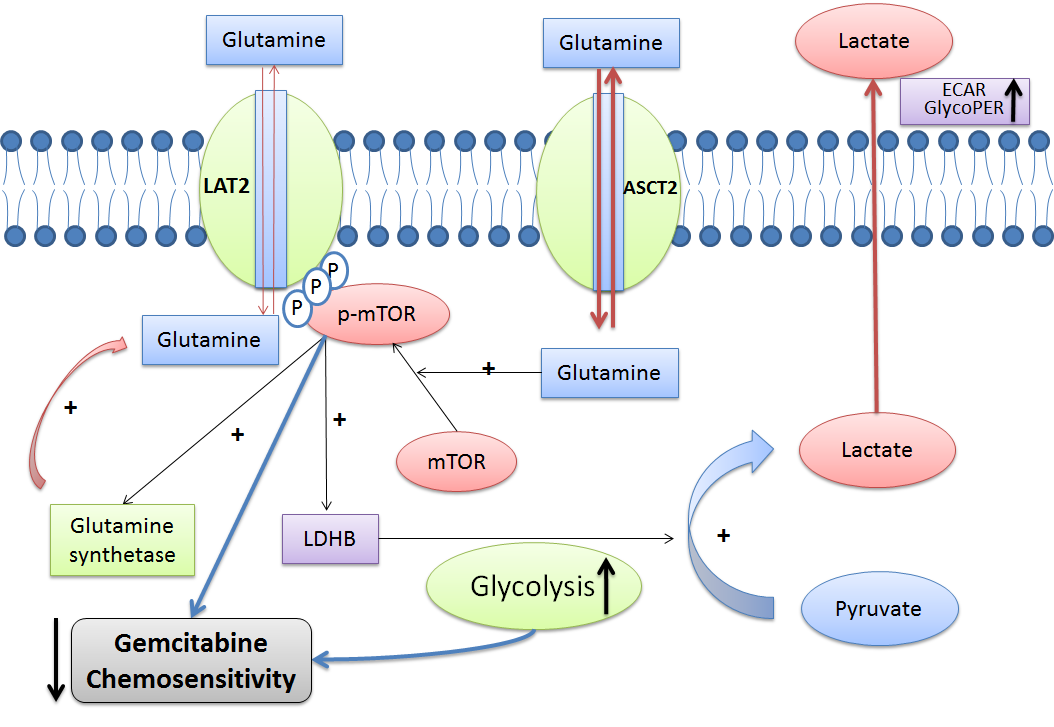

Supplement: Supplementary file 2 — Figure S1. LAT2 promotes pancreatic cancer cell proliferation. (A, C) Overexpression of LAT2 in MIA PaCa-2 and PANC-1 cells promoted cell proliferation. (B, D) LAT2 knockdown suppressed cell proliferation. (*, P < 0.05). Figure S2. LAT2 inhibits pancreatic cancer cell apoptosis. (A, C) LAT2 knockdown in MIA PaCa-2 and PANC-1 cells increased the percentage of apoptotic cells compared with the control. (B, D) Overexpression of LAT2 decreased the percentage of apoptotic cells compared with the control. (E) The Western blotting results revealed that Bax and cleaved caspase-7 were upregulated in MIA PaCa-2 and PANC-1 cells transfected with siLAT2, whereas p21 was downregulated; contrary results were achieved when the cells were transfected with LAT2 OE plasmids. (*, P < 0.05). Figure S3. LAT2 activates glycolysis and alters glutamine metabolism in vivo. The IHC results revealed that the levels of LAT2, LDHB, p-mTORSer2448 and glutamine synthetase were increased in tumors generated from pLVX-PANC-1-LAT2 cells in nude mice compared with those in tumors generated from the control cells, whereas the level of glutaminase was decreased. Figure S4. A high LDHB level is associated with poor prognosis in pancreatic cancer. (A) The Wilcoxon signed rank test was applied to compare the difference in LDHB expression between cancer and paracancerous tissues. (B, C) The expression level of LDHB in 69 pancreatic cancer tissue samples was higher than that in the matched paracancerous tissues (*, P < 0.05). (D, E) Kaplan-Meier survival analysis indicated that the overall survival rate of the high-LDHB and high-LAT2 level group is poorer than that of pancreatic cancer patients with other LDHB and LAT2 statuses (P = 0.0044). Figure S5. LAT2 regulates glutamine-dependent mTOR activation to promote glycolysis and decrease gemcitabine sensitivity. This diagram shows the mechanisms by which LAT2 regulates glycolysis and gemcitabine sensitivity in pancreatic cancer. ASCT2, ASC family transporter [file 13046_2018_947_MOESM2_ESM.zip › Figure S5.tif]
